# Analysis of factors associated with local recurrence after endoscopic resection of gastric epithelial dysplasia: a retrospective study

**DOI:** 10.1186/s12876-020-01293-0

**Published:** 2020-05-12

**Authors:** Min Kyung Back, Hee Seok Moon, In Sun Kwon, Jae Ho Park, Ju Seok Kim, Sun Hyung Kang, Jae Kyu Sung, Eaum Seok Lee, Seok Hyun Kim, Byung Seok Lee, Hyun Yong Jeong

**Affiliations:** 1 Division of Gastroenterology, Department of Internal Medicine, Chungnam National University Hospital, Chungnam National University School of Medicine, 282 Munhwa-ro, Jung-gu, Daejeon, 35015 Korea; 2 Division of Gastroenterology, Department of Internal Medicine, Chungnam National University Hospital, Chungnam National University School of Medicine, 282 Munhwa-ro, Jung-gu, Daejeon, 35015 Republic of Korea; 3 Clinical Trials Center, Chungnam National University Hospital, Daejeon, 34952 Korea

**Keywords:** Gastric dysplasia, Endoscopic treatment, Local recurrence, Endoscopic resection

## Abstract

**Background:**

Endoscopic mucosal resection (EMR) and endoscopic submucosal dissection (ESD) are widely used techniques for the treatment of gastric epithelial dysplasia. Previous studies have compared the clinical outcome of endoscopic resection for early gastric cancer, but few studies have focused on gastric dysplasia alone. This study aimed to evaluate the long-term prognosis following endoscopic procedures for gastric epithelial dysplasia, investigate differences in local recurrence rates according to the treatment modality, and identify risk factors associated with local recurrence.

**Methods:**

In this retrospective study, local recurrence rates and risk factors associated with local recurrence were compared between 599 patients who underwent EMR and 306 who underwent ESD for gastric epithelial dysplasia from January 2011 to December 2015.

**Results:**

The en bloc resection rate (32.2% vs. 100%, *p* < 0.001) and complete resection rate (94.8% vs. 99.0%, *p* = 0.003) were significantly lower in the EMR group than in the ESD group. The local recurrence rate was significantly lower in the ESD group (1.3%) than in the EMR group (4.2%; *p* = 0.026). There was a significantly increased risk of local recurrence, regardless of lesion location or histologic grade, in patients with lesions > 2 cm (*p* = 0.002) or red in color (*p* = 0.03). The ESD group had a significantly lower local recurrence rate, with a higher complete resection rate, than that in the EMR group (*p* < 0.05). In the case of recurrence after endoscopic resection, most of the recurred lesions were removed through additional endoscopic procedures; there was no difference between the two groups (*p* = 0.153).

**Conclusions:**

The complete resection rate was significantly higher, and the local recurrence rate was significantly lower, in patients with gastric epithelial dysplasia treated with ESD. Therefore, ESD should be considered the preferred treatment in patients with lesions > 2 cm or showing redness due to an increased risk of local recurrence and EMR may be possible for low-grade dysplasia that is less than 2 cm without surface changes such as redness, depression and nodularity.

## Background

Gastric adenoma or dysplasia can be defined as a precancerous lesion or an atypical change originating from the stomach epithelium. Approximately 11% of gastric dysplasias are reported to progress to cancer within 4 years. Moreover, 8–59% of gastric dysplasias are associated with gastric cancer [1].

Endoscopic mucosal resection (EMR) and endoscopic submucosal dissection (ESD) are widely used techniques for the treatment of gastric epithelial dysplasia. Because high-grade dysplasia (HGD) is an obvious precancerous lesion, aggressive treatment such as surgical resection or endoscopic treatment is required. However, low-grade dysplasia (LGD) is associated with a relatively low (3–9%) incidence of gastric cancer [2]. Histological examination of resected lesions following endoscopic treatment for dysplasia may lead to an upgrade of the final diagnosis. Therefore, there is a continuing debate as to whether an aggressive LGD treatment or selective treatment of lesions with risk factors for cancer is needed. Therefore, this study aimed to evaluate the long-term prognosis following endoscopic procedures for gastric epithelial dysplasia, to investigate differences in local recurrence rates according to treatment modality, and to evaluate risk factors associated with the local recurrence of dysplasia.

## Methods

### Patients

**Fig. 1:**
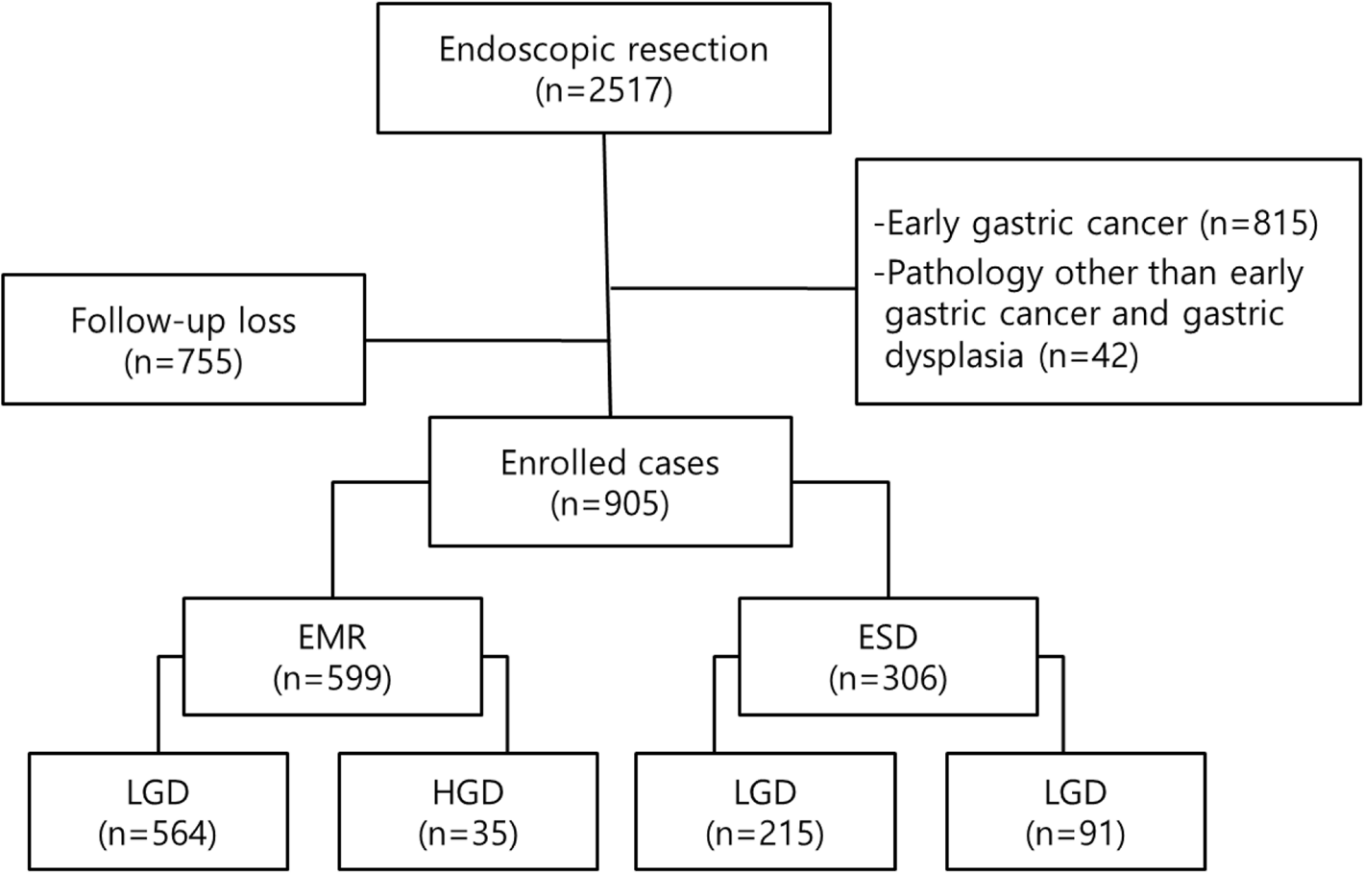
Flowchart of patient enrollment. The clinical outcomes after endoscopic resection of gastric dysplasia in 905 patients were analyzed. EMR, endoscopic mucosal resection; ESD, endoscopic submucosal dissection; HGD, high-grade dysplasia; LGD, low-grade dysplasia

A total of 2517 patients treated with endoscopic resection at Chungnam National University Hospital (CNUH) from January 2011 to December 2015 were screened (Fig. 1). Among these, 857 patients were excluded from the study because they were not diagnosed with gastric dysplasia at the initial endoscopic biopsy (e.g., adenocarcinoma, neuroendocrine tumor, hyperplastic polyp, lipoma, intestinal metaplasia). In addition, 755 patients were excluded because they were not followed up for more than 1 year after endoscopic resection. As a result, a total of 905 patients diagnosed with LGD or HGD by initial endoscopic biopsy were included in the study. All patients underwent endoscopic resection through EMR or ESD, regardless of the lesion size. EMR and ESD were performed in 66.2% (*n* = 599) and 33.8% (*n* = 306) of cases, respectively. Data regarding comorbidities (i.e., hypertension, diabetes mellitus, chronic obstructive pulmonary disease, or chronic kidney disease), smoking history, and alcohol consumption history were collected by medical record review.

### Evaluation of endoscopic features

The surface gross type (i.e., elevated, flat, depressed, or nodularity), color change (i.e., whitish or redness), size, location, atrophic gastritis, and intestinal metaplasia were determined by a review of endoscopic recordings and photographs. Lesion location was classified by dividing the stomach in three equal sections: upper (fundus and upper body), middle (middle and lower body), and lower (angle and antrum). Lesion size was classified as 2.0 cm or > 2.0 cm.

### EMR/ESD techniques

The approach to endoscopic resection for gastric dysplasia was determined by the endoscopists, in consideration of the lesion characteristics, such as size, shape, and location.

During the procedure, midazolam or diazepam was administered intravenously for sedation, and cardiorespiratory functions were monitored. A dual-channel gastroduodenoscope (GIF-ITQ 260 M; Olympus, Japan) was used for EMR and a single-channel gastroduodenoscope (GIF-H260; Olympus) was used for ESD.

**Fig. 2:**
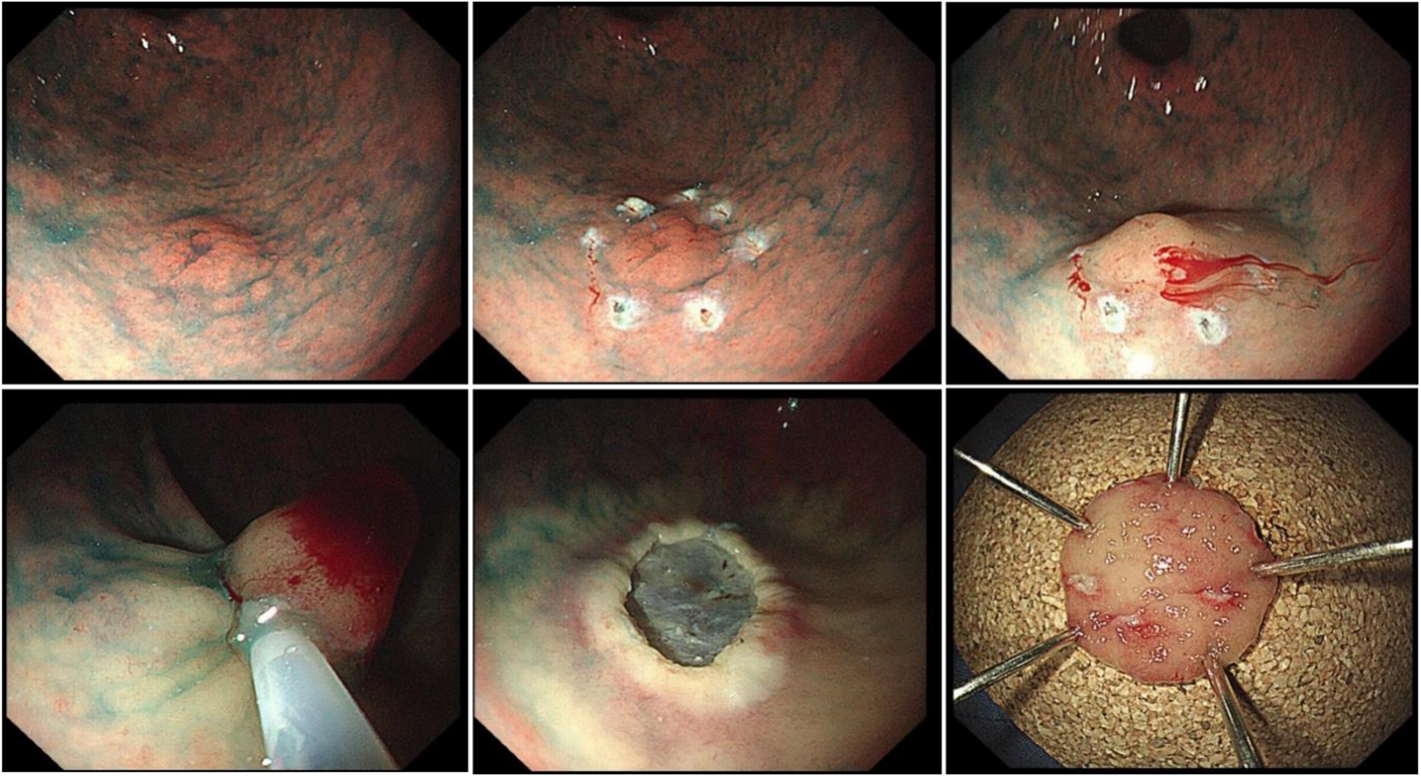
Endoscopic mucosal resection procedure

Before endoscopic resection, 0.1% indigo carmine solution was applied to the lesion to identify its location and margins. After confirming the lesion, areas of the normal mucosa 1–2 mm away from the margin of the lesion were marked with a fixed flexible snare (KachuTechnology Co., Ltd. Korea) or electrosurgical generator (ERBE VIO300D VIO 300D; Erbe, Tübingen, Germany). Then, a saline solution containing diluted epinephrine (1:10,000) was injected into the submucosal layer of the lesion using needle forceps, and the mucosal layer was completely floated from the muscular layer of the lesion. These procedures were the same for both EMR and ESD, but the subsequent steps differed. For EMR, the lesion was pulled using grasping forceps, and en bloc resection was performed by using a snare loop to cover all marked regions at once; if en bloc resection was impossible, a piecemeal resection was performed (Fig. 2). For ESD, an incision was made with a fixed flexed snare outside the marker, and a circumferential incision was performed using an IT knife (single-use electrosurgical knife KD-61 1 L; Olympus). Then, the submucosal layer was dissected until the lesion was completely resected using an IT knife.

**Fig. 3:**
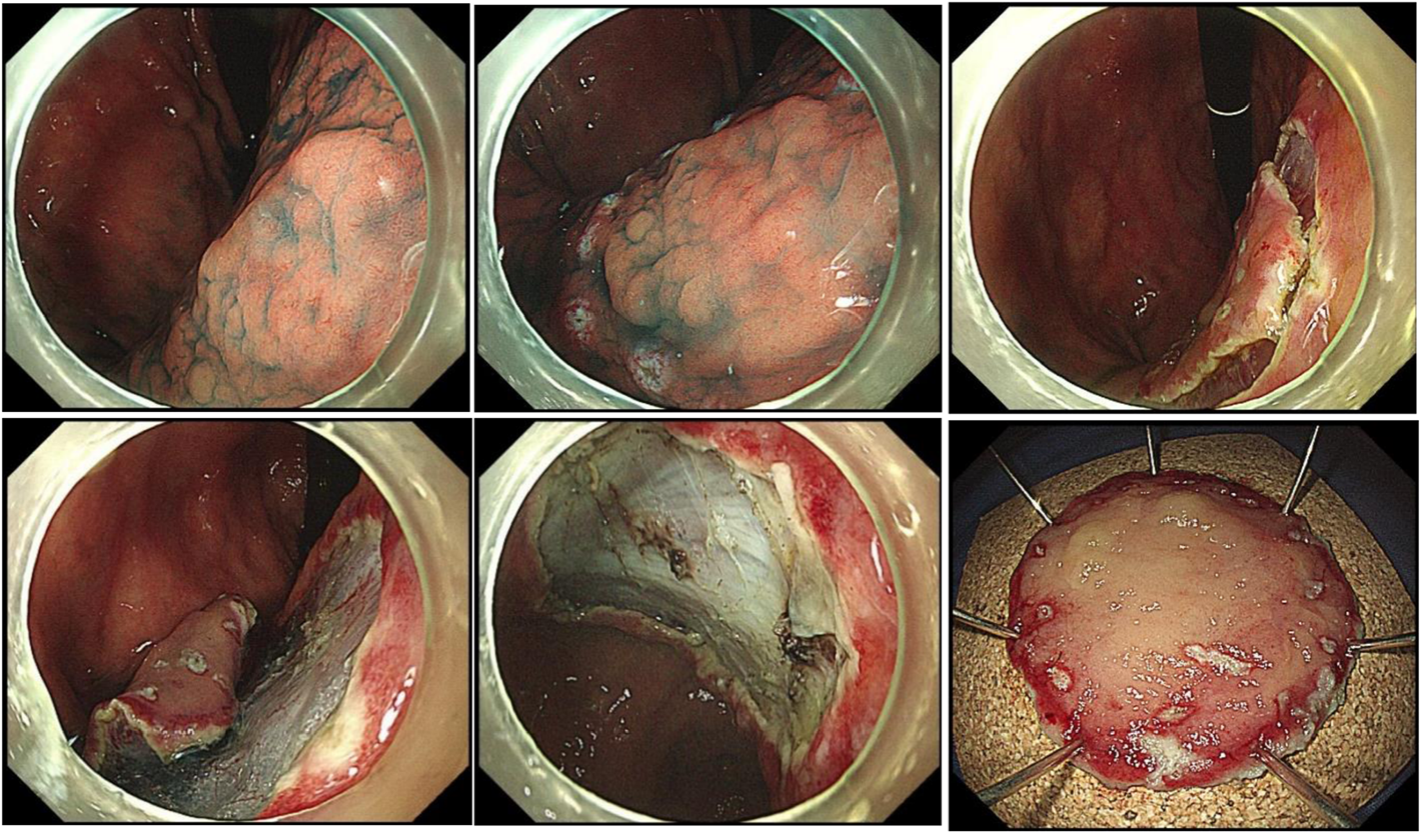
Endoscopic submucosal dissection procedure

During or after endoscopic resection, endoscopic hemostasis was performed for any bleeding or exposed vessels using an IT knife or hemostatic forceps (FD-410LR; Olympus) (Fig. 3). Figure 2 shows the process of en bloc resection of a gastric dysplasia by EMR, and Fig. 3 shows the process of en bloc resection of a gastric dysplasia (approximately 2 cm in size) by ESD**.**

### Definitions

En bloc resection was defined as resection of a lesion in one piece (as opposed to piecemeal resection) [3]. Complete resection was defined as R0 resection in which the resected lesion was pathologically free of dysplasia in the lateral and deep margins. After the procedure, patients were evaluated for complications such as bleeding, perforation, and pyloric stenosis. Delayed bleeding was defined as hematemesis or melena, with a decrease in the hemoglobin level of more than 2 g/dl, requiring endoscopic hemostasis after endoscopic resection [3, 4]. Perforation was defined as direct perforation of the mesenteric fat during endoscopic procedures or free air on abdominal x-ray examination after endoscopic resection [3]. Pyloric stenosis was defined as the occurrence of symptoms such as dyspepsia due to a narrowing of the pyloric ring precluding passage of an endoscopic fiber after endoscopic resection [5].

### Histological analysis

All specimens collected for histological analysis were immediately fixed into paraffin blocks using a neutral buffer with 10% formalin. Paraffin blocks were cut at 2-mm intervals and stained with hematoxylin and eosin to confirm complete resection [2]. The presence of *Helicobacter pylori* was evaluated by the rapid urease test (CLO1 test; Kimberly-Clark, UT) and histologic examination (Wright-Giemsa stain). If any test results were positive, *H. pylori* infection was considered as present. Histologic diagnosis was made by experienced pathologists, in accordance with the Vienna classification of gastric epithelial dysplasia [6].

### Follow-up and confirmation of local recurrence

All patients had their first follow-up endoscopy at 3 or 6 months after endoscopic resection, and annually thereafter. During the follow-up endoscopy, a biopsy was performed when an abnormality was found, such as an overgrowth of the mucosa at the scar of the previous endoscopic resection or a change in color. The results of the histopathologic examination were defined as local recurrence in cases under categories 3–5 of the Vienna classification (i.e., LGD, HGD, or adenocarcinoma).

### Statistical analysis

All statistical analyses were performed using IBM SPSS Statistics version 21 (IBM Corp., Armonk, NY). Statistical significance was set at *p* < 0.05. Group differences (EMR vs. ESD) in baseline characteristics were evaluated using Pearson’s chi-squared test for categorical variables. A logistic regression model was used to analyze factors affecting local recurrence. Significant univariate factors (p < 0.05) were examined using a multivariate Cox proportional hazards regression model to identify the independent factors associated with local recurrence. Odds ratios (OR) and 95% confidence intervals (CI) were calculated to estimate the relative risks of local recurrence. The Kaplan-Meier method was used to analyze the cumulative rate of local recurrence.

## Results

### Clinicopathological characteristics

Endoscopic resection for gastric epithelial dysplasia was performed in 905 patients. These patients were divided into an EMR group (*n* = 599) and an ESD group (*n* = 306). Table 1 shows a comparison of the characteristics and endoscopic findings between the groups. No significant group differences in age, sex, comorbidities, social history (alcohol or smoking), and atrophic gastritis were found. Lesions 2 cm in size were treated significantly more frequently with EMR than with ESD (*p* < 0.05). Both groups showed more lesions in the antrum than in other areas, but no statistically significant differences were noted. A depressed or reddish lesion was treated significantly more frequently with ESD than with EMR (*p* < 0.05). Moreover, the groups significantly differed in the incidences of intestinal metaplasia and *H. pylori* infection (p < 0.05). In addition, when the pathology results were HGD, the lesions were more likely to be treated with ESD than with EMR (p < 0.05).

**Table 1:** Baseline characteristics and endoscopic findings in the EMR and ESD groups

	EMR (n = 599)	ESD (*n* = 306)	*P* value
Age (yr), mean SD	65.19 ± 8.781	64.50 ± 8.099	0.249
Sex (%)			0.694
Male	432 (72.1)	225 (73.5)	
Female	167 (27.9)	81 (26.5)	
Comorbidity (%)			
DM	91 (66.9)	45 (33.1)	0.922
HTN	215 (63.8)	122 (36.2)	0.246
COPD	16 (69.6)	7 (30.4)	0.826
CKD	12 (57.1)	9 (42.9)	0.362
Alcohol	147 (64.8)	80 (35.2)	0.627
Smoking	123 (64.1)	69 (35.9)	0.498
Location (%)			0.632
Upper (fundus, UB)	117 (19.5)	55 (18.0)	
Mid (MB, LB)	181 (30.2)	87 (28.4)	
Lower (angle, antrum)	301 (50.3)	164 (53.6)	
Size (%)			0.006
0–2 cm	563 (94.0)	271 (88.6)	
> 2 cm	36 (6.0)	35 (11.4)	
Color change (%)			0.000
Redness	188 (31.4)	188 (61.4)	
Whitish	411 (68.6)	118 (38.6)	
Gross type (%)			0.000
Elevated	220 (36.7)	50 (16.3)	
Flat	175 (29.2)	78 (25.5)	
Depressed	93 (15.5)	129 (42.2)	
Nodularity	111 (18.5)	49 (16.0)	
Atrophic change (%)			0.684
Closed type	555 (92.7)	286 (93.5)	
Open type	44 (7.3)	20 (6.5)	
Intestinal metaplasia (%)			
Yes	201 (33.6)	137 (44.8)	0.001
*Helicobacter pylori* (%)			
Positive	70 (11.7)	66 (21.6)	0.000
Endoscopic biopsy (%)			0.000
LGD (%)	564 (94.2)	215 (70.3)	
HGD (%)	35 (5.8)	91 (29.7)	

*CKD* Chronic kidney disease, *COPD* Chronic obstructive pulmonary disease, *DM* Diabetes mellitus, *EMR* Endoscopic mucosal resection, *ESD* Submucosal dissection, *HGD* High-grade dysplasia, *HTN* Hypertension, *LB* Lower body, *LGD* Low-grade dysplasia, *MB* Middle body, *SD* Standard deviation, *UB* Upper body

### Therapeutic outcomes

The clinical outcomes of EMR and ESD are shown in Table 2. Complete and en bloc complete resection rates were higher in the ESD group than in the EMR group (complete resection rates, 99.0% vs. 94.8%, respectively; *p* = 0.003; en bloc resection rates, 100% vs. 32.2%, respectively; *p* < 0.001). No patients developed perforation after the endoscopic resection. Further, no significant differences in the incidence rate of delayed bleeding after endoscopic resection were observed between the two groups. Bleeding after ESD was frequently observed within 3 days, and bleeding after EMR was usually observed within 2 weeks. The bleeding after endoscopic resection was treated with endoscopic hemostasis through HSE injection, hemoclipping and / or APC. Pyloric stenosis occurred in only 2 of the patients who underwent EMR, without a statistically significant difference between the two groups. Pyloric stenosis after endoscopic treatment was treated with endoscopic balloon dilatation.

**Table 2:** Clinical outcomes of EMR and ESD

	EMR (n = 599)	ESD (n = 306)	P value
En bloc resection (%)	193 (32.2)	306 (100)	0.000
Complete resection (%)	568 (94.8)	303 (99.0)	0.003
Complication (%)			0.065
Bleeding	20 (3.3)	21 (6.9)	
Perforation	0	0	
Stricture	2 (0.3)	0 (0.0)	
Post-procedure histopathologic result (%)			0.000
Other	31 (5.2)	4 (1.3)	
LGD	469 (78.3)	175 (57.2)	
HGD	46 (7.7)	76 (24.8)	
Adenocarcinoma	53 (8.8)	51 (16.7)	
Median follow-up duration	43.29 ± 20.47	32.21 ± 17.60	
Local recurrence (%)	25 (4.2)	4 (1.3)	0.026
Cumulative incidence	25.02 ± 18.84	14.56 ± 5.653	0.286

*EMR* Endoscopic mucosal resection, *ESD* Submucosal dissection, *HGD* High-grade dysplasia, *LGD* Low-grade dysplasia

**Fig. 4:**
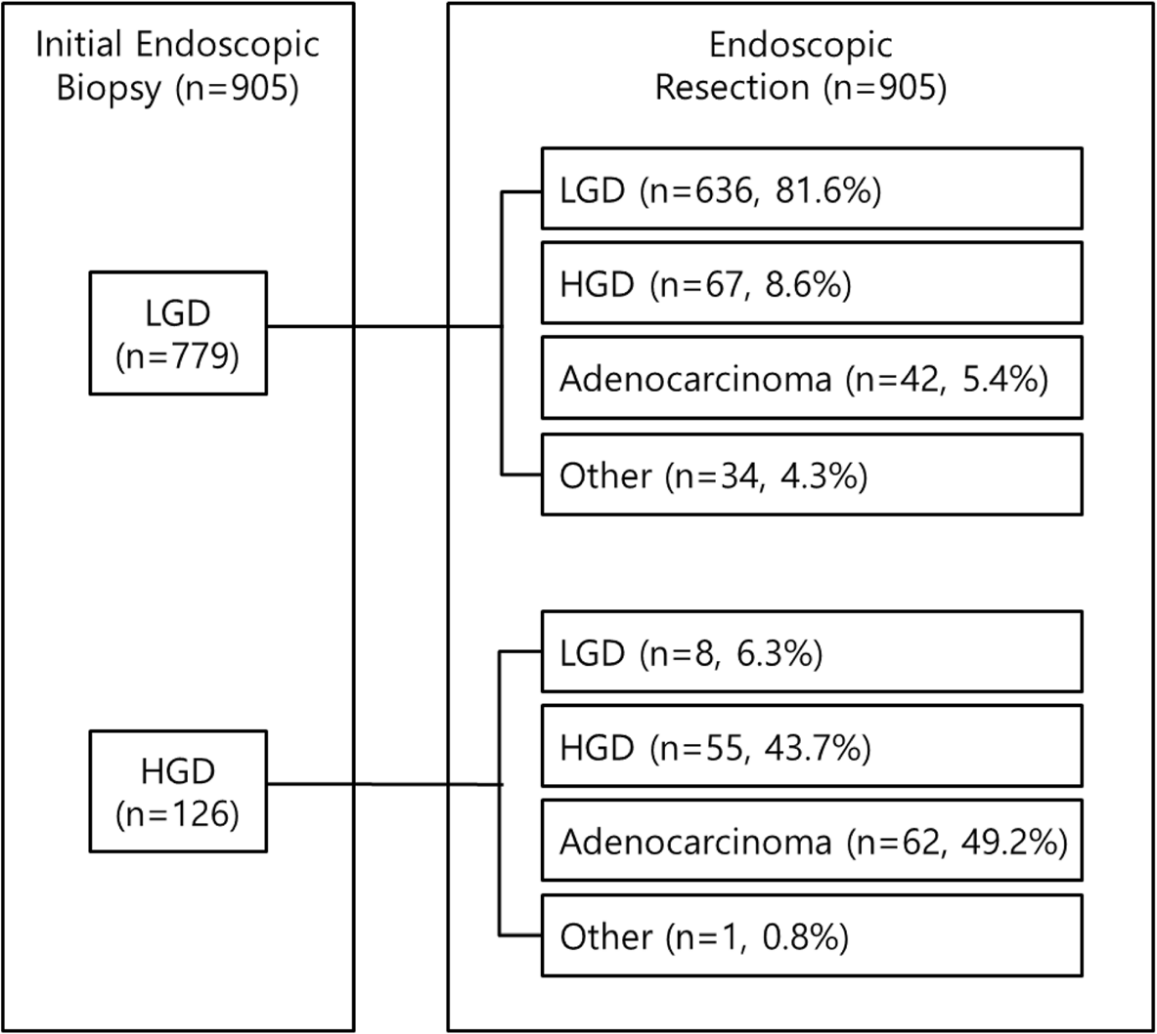
Pathological changes after endoscopic resection. Other: No residual adenomatous lesion, regenerative atypia, or chronic active gastritis with intestinal metaplasia. HGD, high-grade dysplasia; LGD, low-grade dysplasia

Figure 4 shows the histological changes before and after endoscopic resection. Of the 779 LGD patients, 109 (14%) were histologically upgraded to HGD or adenocarcinoma after endoscopic resection. Of the 126 HGD patients, 62 (49.2%) were histologically upgraded to adenocarcinoma after endoscopic resection. Eight of the patients diagnosed with HGD were downgraded to LGD after endoscopic resection (about 6.3%). In addition, no residual dysplasia was found on endoscopy after endoscopic resection in 35 patients (Table 2). In both groups, the final pathologic results after endoscopic resection showed a higher ratio of LGD compared to those of HGD and adenocarcinoma (EMR vs. ESD; 78.3% vs. 57.2%); however, ratios of HGD and adenocarcinoma were higher in the EMR group than in the ESD group.

**Fig. 5:**
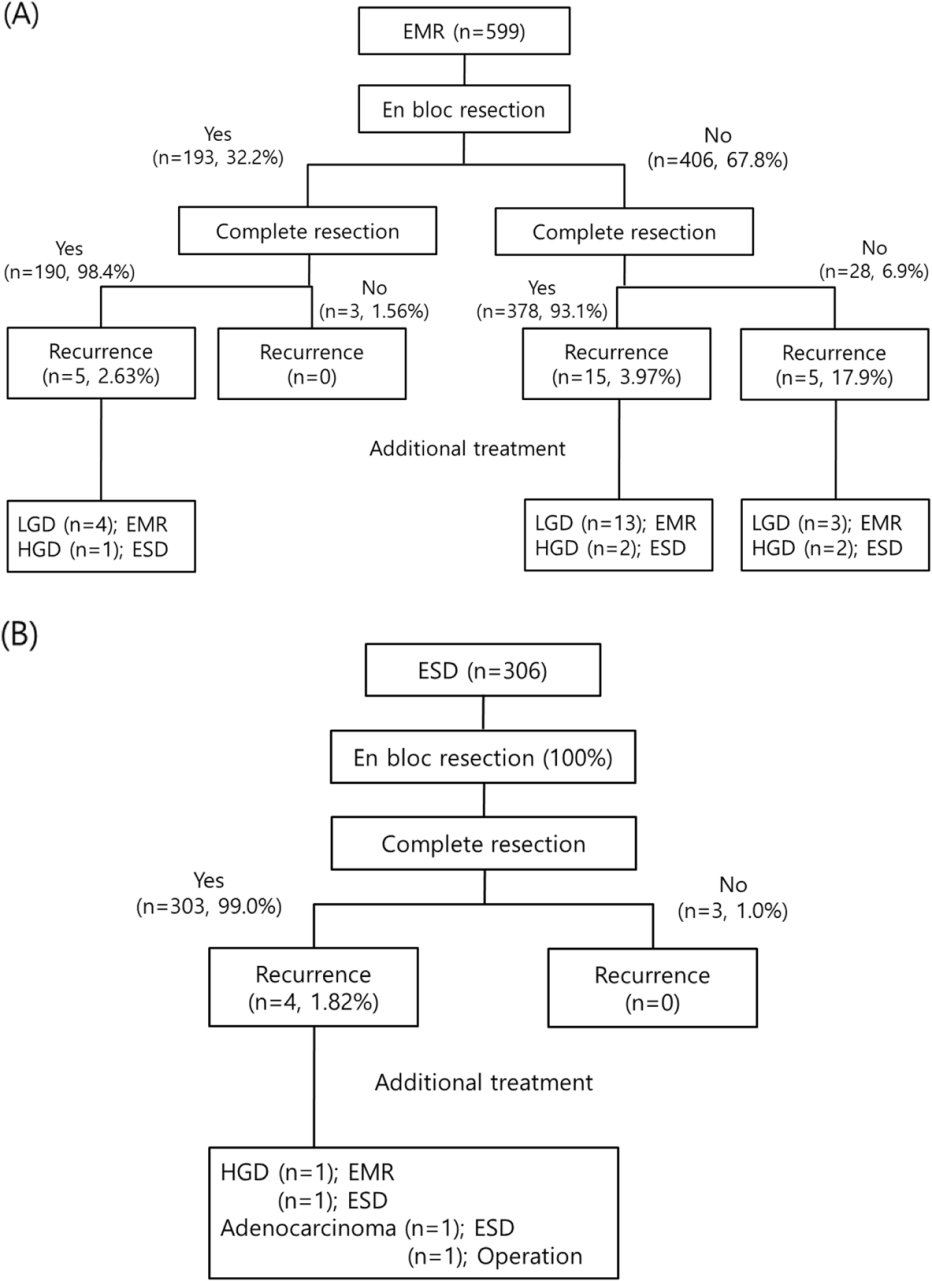
Clinical course after endoscopic resection. **a** In the EMR group, the piecemeal resection rate was relatively high, and local recurrence occurred in 17.9% of patients who did not undergo both en bloc and complete resection. **b** In the ESD group, local recurrence occurred in about 1.82% of patients with both en bloc and complete resection, which is lower than the rate in the EMR group. EMR, endoscopic mucosal resection; ESD, endoscopic submucosal dissection; HGD, high-grade dysplasia; LGD, low-grade dysplasia

**Fig. 6:**
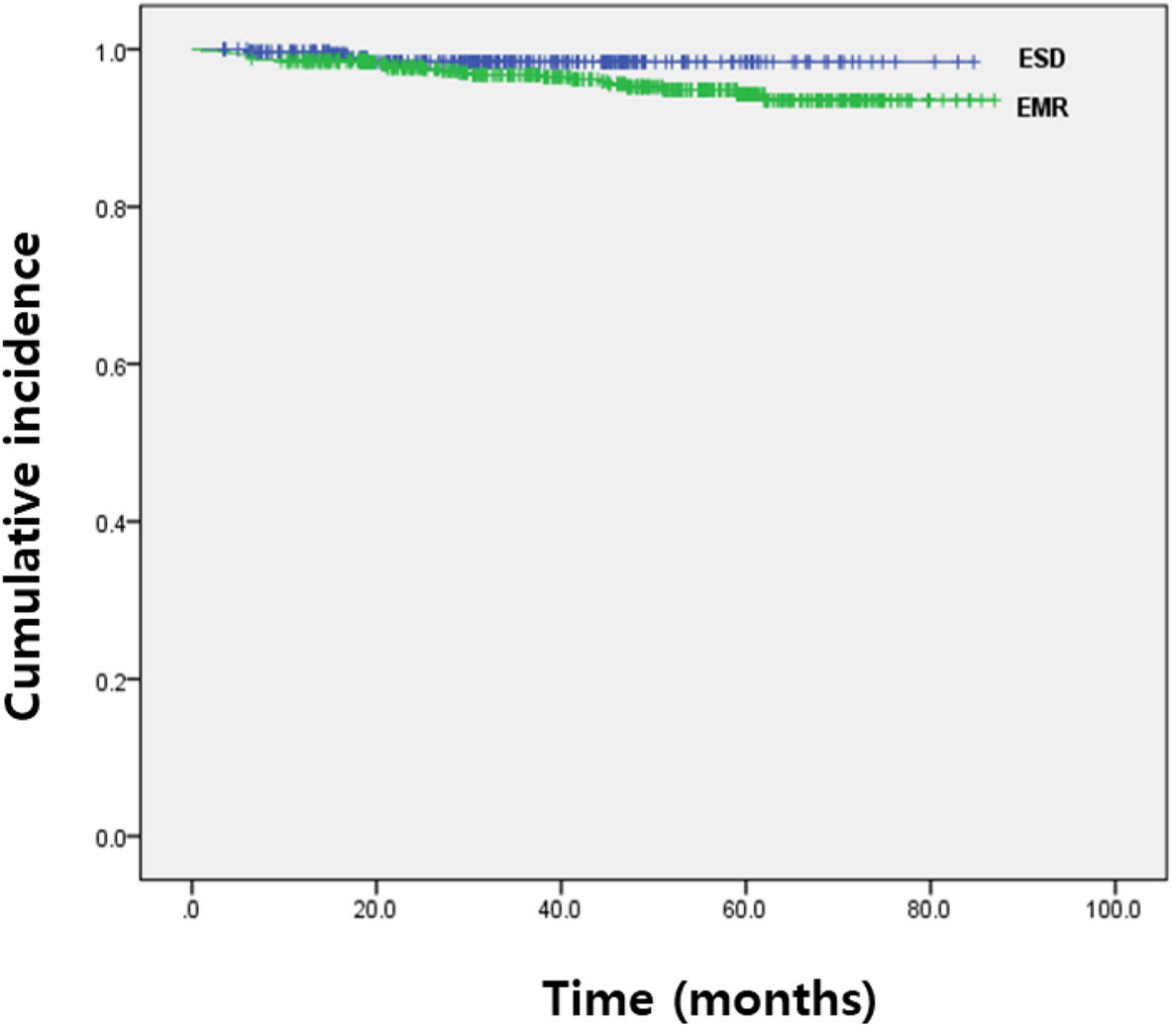
Cumulative incidence of local recurrence after EMR or ESD. Kaplan-Meier curves show the trend of the development of local recurrence after endoscopic resection. EMR, endoscopic mucosal resection; ESD, endoscopic submucosal dissection

Figure 5 shows the en bloc resection and complete resection rates in patients with local recurrence. Local recurrence occurred in 20 of 406 patients who underwent piecemeal resection (about 4.9%) and in 9 of 499 patients who underwent en bloc resection (about 1.8%). The local recurrence rate after endoscopic resection was significantly higher in the EMR group than in the ESD group (*p* = 0.026). One patient who experienced relapse and was finally diagnosed with adenocarcinoma underwent surgical treatment, and the rest underwent complete resection with additional endoscopic treatment for local recurrence (Fig. 5) and there was no difference between the two group (EMR vs. ESD; 100% (25/25) vs. 75% (3/4); *p* = 0.153). There were no disease-related death among the 29 patients. The Kaplan-Meier analysis showed no significant difference in the cumulative incidence between the two groups (*p* = 0.286), with a median follow-up duration of 39.55 ± 20.23 months (Table 2, Fig. 6).

### Risk factors for local recurrence by logistic regression analysis

Table 3 shows the results of the univariate logistic regression analysis of the risk factors for local recurrence. The local recurrence rate after endoscopic resection was higher for lesions > 2 cm than for those 2 cm (OR 4.037, *p* = 0.002). In addition, the local recurrence rate was significantly lower when en bloc resection and complete resection were performed (en bloc resection, OR 0.011, *p* = 0.011; complete resection, OR 0.164, *p* = 0.001). In terms of the endoscopic resection method, the local recurrence rate was lower with ESD than with EMR (OR 0.304, *p* = 0.028). Table 4 shows the results of the multivariate logistic regression analysis of the risk factors for local recurrence. The local recurrence rate was higher in cases with larger lesions, and was lower when complete resection or ESD was performed.

**Table 3:** Univariate logistic regression analysis of the risk factors for local recurrence

	Univariate	
	P value	OR (95% CI)
Size		
0–2 cm vs. > 2 cm	0.002	4.037 (1.661–9.809)
Color change		
Redness	0.716	1.148 (0.546–2.416)
Gross type		
Elevated	0.292	
Elevated vs. flat	0.871	0.913 (0.303–2.753)
Elevated vs. depressed	0.710	1.223 (0.423–3.541)
Elevated vs. nodularity	0.117	2.239 (0.817–6.135)
Atrophic change		
Closed type	0.450	2.170 (0.290–16.211)
Intestinal metaplasia		
Yes	0.220	1.590 (0.758–3.337)
*Helicobacter pylori*		
Yes	0.111	0.196 (0.026–1.453)
Endoscopic biopsy		
LGD vs. HGD	0.113	2.024 (0.846–4.842)
En bloc resection		
Yes	0.011	0.354 (0.160–0.787)
Complete resection		
Yes	0.001	0.164 (0.059–0.461)
Complication		
Yes	0.998	0.000
Pathologic result of post-procedure		
Other^a^	0.424	
Other vs. LGD	0.983	0.978 (0.127–7.541)
Other vs. HGD	0.503	2.070 (0.246–17.414)
Other vs. adenocarcinoma	0.993	1.010 (0.102–10.036)
Procedure		
ESD	0.028	0.304 (0.105–0.882)

*CI* Confidence intervals, *EMR* Endoscopic mucosal resection, *ESD* Submucosal dissection, *HGD* High-grade dysplasia, *LGD* Low-grade dysplasia, *OR* Odds ratio

^a^Other includes the cases with no residual adenomatous lesions, regenerative atypia, or chronic gastritis with intestinal metaplasia on pathological examination after endoscopic resection

**Table 4:** Multivariate logistic regression analysis of the risk factors for local recurrence

	Multivariate	
	P value	OR (95% CI)
Size		
0–2 cm vs. > 2 cm	0.006	3.893 (1.489–10.180)
Complete resection		
Yes	0.030	0.292 (0.096–0.887)
Procedure		
ESD	0.027	0.293 (0.098–0.872)

*CI* Confidence intervals, *ESD* Submucosal dissection, *OR* Odds ratio

## Discussion

Previous studies comparing clinical outcomes according to endoscopic resection methods have focused mostly on early gastric cancer, and there have been few studies on gastric dysplasia alone. Therefore, this study was carried out to compare the local recurrence rates for EMR and ESD and to identify the risk factors of local recurrence. The results showed that the complete resection rate was significantly higher, and the local recurrence rate was significantly lower, in patients with gastric epithelial dysplasia treated with ESD rather than with EMR.

According to the National Cancer Information Center of Korea, as of 2016, stomach cancer had the highest incidence, at 35%, compared to that for other solid cancers [7]. In Korea, gastroduodenoscopy during a health checkup is recommended every 2 years, starting at the age of 40 years. Therefore, the diagnosis of gastric dysplasia, as well as early gastric cancer, is increasing. According to the Correa hypothesis, gastric dysplasia is a precancerous lesion that progresses from gastric atrophy and intestinal metaplasia to adenocarcinoma through hypoplasia or dysplasia [8]. However, a previous study on the natural course of gastric dysplasia showed that LGD progressed to adenocarcinoma at a relatively low rate of about 0–23%, while HGD showed a higher progression rate of about 10–81% [9]. In a recent study that followed patients with gastric dysplasia for 7 years, only 7.8% of patients with LGD cases progressed to cancer during follow-up, while 63.6% of those with HGD cases progressed to cancer [10]. In the present study, the final pathological diagnosis was not changed after endoscopic resection in 81.6% of LGD patients, but 49.2% of HGD patients were diagnosed with adenocarcinoma after endoscopic resection (Fig. 2). Thus, there is no question that HGD requires endoscopic resection or surgical treatment because of its potential for cancer progression and the coexistence of cancer cells. In contrast, LGD has a relatively low risk of malignant transformation, and spontaneously regresses in 32–59% of patients in previous studies [11–16]. The American Society for Gastrointestinal Endoscopy and the British Society of Gastroenterology guidelines recommend endoscopic resection for gastric dysplasia of any size, if possible [17, 18]. The European guidelines also recommend grading and resecting dysplasia in patients with visible endoscopic lesions. If there is no visible endoscopic lesion, it is necessary to confirm the lesion by magnification chromoendoscopy and/or narrow-band imaging. If the lesion is confirmed, a biopsy is performed, and if the diagnosis is LGD, follow-up endoscopy should be performed within 12 months [19]. In Korea, because LGD may sometimes progress to cancer, endoscopic resection is performed, unless it is impossible because of advanced age or comorbid disease.

Gastric dysplasia is mainly treated by endoscopic resection, but argon plasma coagulation (APC) is also used. Several studies have shown that the removal of gastric dysplasia through APC curettage is a good option because of the short hospitalization period, low medical costs, and low complication rates [20, 21]. However, APC is effective for only relatively small-sized LGD lesions (2.0 cm), and the local recurrence rate is higher than that with other endoscopic methods [21]. In addition, since the tissue cannot be collected, determining the final histological diagnosis of the lesion is difficult. Therefore, ESD and EMR are considered as standard treatments for gastric dysplasia, and are used as an additional method to remove remaining lesions after endoscopic resection.

In comparison with conventional EMR, ESD requires a long treatment time and advanced operator skills, and has disadvantages associated with complications such as perforation. However, the advantages of ESD are the en bloc resection of large lesions, high complete resection rate, and low local recurrence rate [9, 22–26].

In a 2010 study on the predictive factors for local recurrence after endoscopic resection for early gastric cancer, a larger lesion size and treatment with EMR were associated with increased an incomplete resection rate, and had a significant impact on local recurrence [27]. Data from this study showed that en bloc resection can be an important predictor of local recurrence. Histologic type, comorbidities, location of the lesion, color of the lesion, gross type, and presence of *H. pylori* infection were not correlated with local recurrence; only the size of the lesion and an incomplete resection were risk factors of local recurrence. In this study related to recurrence after resection of gastric dysplasia, most cases of relapse after endoscopic treatment could be treated with additional endoscopic resection (97%, 28/29) Also, this shows that even for the lesions resected with EMR, the most of them could achieve complete eradication on a subsequent endoscopy by repeating the EMR.

The present study has several limitations. First, the study was retrospective in nature and was conducted at a single center. Endoscopic resection was performed by four endoscopists, and EMR or ESD procedures were chosen according to their subjective judgment, in the absence of definitive treatment guidelines for gastric dysplasia. The individual endoscopists may also have influenced clinical outcomes; although they were trained at the same institution, they differed in experience and preferences, such as the use of particular knives and electrocoagulation modes. Thus, the en bloc resection rate may vary depending on the endoscopist. Therefore, multicenter and prospective studies are needed to confirm our results. Second, the duration of follow-up was not constant. We analyzed data from patients who underwent endoscopic resection between January 2011 and December 2015 and were followed for at least 1 year after the procedure. Therefore, our observation periods ranged between 7 (long-term) and 3 years (short-term). Third, in the present study, although we investigated *H. pylori* infection and eradication treatments, the effects of the presence or absence of *H. pylori* and eradication were not clear. Thus, further studies are needed to determine their association with local recurrence. Fourth, accurate tumor size measurement is critical for selecting proper candidates for endoscopic resections (ER) of gastric neoplasia. However, size discrepancy between endoscopic size and pathologic size often occurs during ER for gastric tumor.

One strength of the present study is its focus on the endoscopic resection of gastric dysplasia, which is a pre-cancerous lesion, and its determination of the local recurrence rate according to the endoscopic resection method. Furthermore, the discrepancy in the pathological diagnosis before and after endoscopic resection of gastric dysplasia was investigated.

## Conclusions

The present study showed that approximately 49.2% of HGD cases were upgraded histologically to adenocarcinoma after endoscopic resection. In addition, local recurrence rates were higher in patients with lesions > 2 cm, histologically incomplete resection. Therefore, in cases of gastric dysplasia with HGD or a lesion > 2 cm, ESD is preferred to EMR due to its higher incomplete resection rate, which can help prevent local recurrence and also EMR may be possible for low-grade dysplasia that is less than 2 cm without surface changes such as redness, depression and nodularity.

## Data Availability

The datasets used and analyzed in the current study are available from the corresponding author on reasonable request.

## Abbreviations

APC: Argon plasma coagulation
EMR: Endoscopic mucosal resection
ESD: Endoscopic submucosal dissection
HGD: High-grade dysplasia
LGD: Low-grade dysplasia
